# Discernment

**Published:** 1878-12

**Authors:** 


### *DISCERNMENT.*

There is no man but thinks *his* powers of discernment are superior to those of any other person. He sees faults in others which he thinks they do not see in him. The truth is, that it is just as difficult to conceal our unpleasant peculiarities from those who care to find them out as it would be for a giant to hide his burly form behind a ladder. A generous man is often embarrassed in his efforts to seem blind to the faults of others. He may be the honored guest at the home of luxury and seeming refinement; but if there is a skeleton in that house, it is set before him by revelations unconsciously made. Is the husband and father a tyrant? He reveals the fact by an unguarded word or a furtive glance. But he is at home but little, and his peculiarities *can* be endured while he is there. The most unpleasant and unendurable disturber of domestic peace, when she does appear in that *role*, is the female element. It is seldom the care-worn, long-suffering mother, whose heart is chastened and saddened by cares often cruelly thrust upon her; it is far more likely to be a daughter, a child spoiled by over-indulgence, or one whose sweetness has turned to vinegar by the ferment of disappointed hopes. She becomes the worst of tyrants, because she is always on the alert for an opportunity to display her authority. She opposes whatever others propose. Her mother stands in awe of her, and her sisters tremblingly submit to her will for the sake of a peace which is dearly purchased, because of short duration. Does she deceive any one? Does she suppose that an assumed gentleness or a mask of smiles will conceal the deformity visible through the "torn vail?" The attrition of such a temper furrows not alone the face, but the heart, making her friendship undesirable, and as the shadows of life lengthen, she finds herself alone and desolate. Every individual, every family, has trials enough that are unavoidable, without these added miseries. "Home" is a sacred word in its true significance. Evil must be the spirit that would mar the peace and love and joy that have no other dwelling place, and deprive our later years of those cherished remembrances which should have clustered there.

